# Assessment of Gene Variant Amenability for Pharmacological Chaperone Therapy with 1-Deoxygalactonojirimycin in Fabry Disease

**DOI:** 10.3390/ijms21030956

**Published:** 2020-01-31

**Authors:** Jan Lukas, Chiara Cimmaruta, Ludovica Liguori, Supansa Pantoom, Katharina Iwanov, Janine Petters, Christina Hund, Maik Bunschkowski, Andreas Hermann, Maria Vittoria Cubellis, Arndt Rolfs

**Affiliations:** 1 Translational Neurodegeneration Section “Albrecht-Kossel“, Department of Neurology, University Medical Center Rostock, 18147 Rostock, Germany; chiara.cimmaruta@med.uni-rostock.de (C.C.); Supansa.Pantoom@med.uni-rostock.de (S.P.); katharina.iwanov@med.uni-rostock.de (K.I.); janine.petters@med.uni-rostock.de (J.P.); christina.hund@med.uni-rostock.de (C.H.); andreas.hermann@med.uni-rostock.de (A.H.); 2 Center for Transdisciplinary Neurosciences Rostock (CTNR), University Medical Center Rostock, University of Rostock, 18147 Rostock, Germany; 3 Dipartimento di Scienze e Tecnologie Ambientali, Biologiche e Farmaceutiche, Università degli Studi della Campania “Luigi Vanvitelli”, 81100 Caserta, Italy; lud.liguori@gmail.com; 4 Institute of Biomolecular Chemistry, CNR, 80078 Pozzuoli, Italy; cubellis@unina.it; 5 Centogene AG, 18055 Rostock, Germany; Maik.Bunschkowski@centogene.com (M.B.); Arndt.Rolfs@centogene.com (A.R.); 6 German Center for Neurodegenerative Diseases (DZNE) Rostock/Greifswald, 18147 Rostock, Germany; 7 Department of Biology, University Federico II, 80126 Naples, Italy; 8 University Medical Center Rostock, University of Rostock, 18057 Rostock, Germany

**Keywords:** Lysosomal storage disorders, pharmacological chaperones, method comparison study, personalized medicine

## Abstract

Fabry disease is one of the most common lysosomal storage disorders caused by mutations in the gene encoding lysosomal α-galactosidase A (α-Gal A) and resultant accumulation of glycosphingolipids. The sugar mimetic 1-deoxygalactonojirimycin (DGJ), an orally available pharmacological chaperone, was clinically approved as an alternative to intravenous enzyme replacement therapy. The decision as to whether a patient should be treated with DGJ depends on the genetic variant within the α-galactosidase A encoding gene (*GLA*). A good laboratory practice (GLP)-validated cell culture-based assay to investigate the biochemical responsiveness of the variants is currently the only source available to obtain pivotal information about susceptibility to treatment. Herein, variants were defined amenable when an absolute increase in enzyme activity of ≥3% of wild type enzyme activity and a relative increase in enzyme activity of ≥1.2-fold was achieved following DGJ treatment. Efficacy testing was carried out for over 1000 identified *GLA* variants in cell culture. Recent data suggest that about one-third of the variants comply with the amenability criteria. A recent study highlighted the impact of inter-assay variability on DGJ amenability, thereby reducing the power of the assay to predict eligible patients. This prompted us to compare our own α-galactosidase A enzyme activity data in a very similar in-house developed assay with those from the GLP assay. In an essentially retrospective approach, we reviewed 148 *GLA* gene variants from our former studies for which enzyme data from the GLP study were available and added novel data for 30 variants. We also present data for 18 *GLA* gene variants for which no data from the GLP assay are currently available. We found that both differences in experimental biochemical data and the criteria for the classification of amenability cause inter-assay discrepancy. We conclude that low baseline activity, borderline biochemical responsiveness, and inter-assay discrepancy are alarm signals for misclassifying a variant that must not be ignored. Furthermore, there is no solid basis for setting a minimum response threshold on which a clinical indication with DGJ can be justified.

## 1. Introduction

Fabry disease (FD; MIM# 301500) is a rare X–linked lysosomal storage disorder caused by mutations in the *GLA* gene encoding for the lysosomal enzyme α-galactosidase A (α-Gal A, E.C. 3.2.1.22). Pathological changes in the gene and its encoded protein result in a complete cellular absence or insufficiency of α-Gal A enzyme activity. The consequence is a cellular and microvascular dysfunction with multiple organ involvement [1]. The resulting storage of complex sphingolipids in the lysosomes, mainly globotriaosylceramide (Gb3) and its metabolite globotriaosylsphingosine (lyso-Gb3) serve as biomarkers in the diagnosis of FD [2] and are believed to play a major role in disease pathophysiology [3].

Clinical FD manifestation involves acroparesthesia, abdominal pain and fever, angiokeratomas, cornea verticillata, decreased ability to perspire, proteinuria, and progressive renal insufficiency. Considerable morbidity in patients with FD is due to kidney failure, cardiac disease, and stroke in the third to fifth decade of life [4,5,6]. However, a broad heterogeneous symptom spectrum can be observed, which is largely associated with the genotype [7].

To date, more than 1000 mostly private *GLA* gene variants were found related to FD [8]. A majority of approximately 60% of the variants are missense mutations associated with single amino-acid substitutions [9]. Enzyme replacement therapy (ERT) can principally be administered to all FD patients regardless of the underlying *GLA* gene constitution. However, the benefit of ERT is disadvantaged by a number of limitations such as insufficient penetration of relevant tissues [10] and an immune response that can lead to the formation of neutralizing immunoglobulin G (IgG) antibodies [11]. Therefore, the orally available pharmacological chaperone 1-deoxygalactonojirimycin (DGJ or migalastat, trade name Galafold^®^ [12]) was recently developed as an alternative to ERT, but is suitable only for patients carrying biochemically responding gene variants. Typically, variants with residual enzyme activity are likely to respond to chaperone treatment at a higher level [13]. Nevertheless, even gene variants that severely affect enzyme activity can be classified as so-called “amenable”. In addition to the missense variants, these may include nonsense variants near the carboxyl terminus, in-frame small deletions and insertions, and variants with more than one nucleotide exchange on the same allele [14]. A large number of studies concerned the assessment of variant α-Gal A enzyme activity in different cell culture systems. It was found that inter-assay discrepancies in residual activity and DGJ responsivity of the variants persist [15]. During the clinical phase 3 study, a standardized good laboratory practice (GLP)-validated human embryonic kidney cell-based in vitro assay was established to identify DGJ amenability of *GLA* gene variants [14], and it is currently the only approved method for this assessment. A very recent study stressed a significant inter-assay variability between the GLP-validated assay and an in-house assay adapted to it [16]. Due to the impact of this study for physicians, patients, and the relatives of patients, we felt that this study called on our own recent experience with further mutation data in order to contribute to the important topic of amenability of *GLA* gene variants. Thus, we comparatively analyzed the results from our in-house *GLA* gene variant amenability assessment with the GLP study data for reproducibility of enzymatic data and DGJ amenability classification of 178 *GLA* variants.

## 2. Results

Before the 178 datasets of the GLP-validated assay were compared with our in-house assay, the following 10 *GLA* gene variants from previous articles [7,13] were reexamined according to the in-house protocol to evaluate the robustness and reproducibility of the assay: M42V, N139S, G183V, N215S, S247P, L268S, L310F, S345P, R356Q, and G360C. Differences in the reexamination are shown in Table A1 (Appendix A). Herein, one variant, L310F, changed category from non-amenable to amenable in accordance with the GLP-validated study and another former study [17]. Furthermore, a strong linear correlation of baseline activity and activity after DGJ treatment was obtained (Pearson *r* = 0.9484, *p* < 0.0001; *r* = 0.8864, *p* = 0.0006). However, there was no correlation with the DGJ-induced activity change (Pearson *r* = 0.01734, not significant), which can probably be explained by the small case size and the significantly different results for the three variants N139S, L310F (category switch), and R356Q.

### 2.1. Global Description of the Investigated Gene Variants in the In-House Assay

Among the 178 gene variants implemented in the present study, 88 were classified amenable and 90 were classified non-amenable by our in-house assay using our amenability criteria (Table 1). Amenability classification requires an absolute increase in α-Gal A ≥ 5% of wild type (WT) or a relative increase in α-Gal A activity ≥ 1.5-fold above baseline plus a minimum of 5% activity (%WT) after incubation with 20 μM DGJ. Of the 88 amenable variants, all showed the required increase in absolute enzyme activity of 5%. Only 58 of these showed the 1.5-fold relative increase compared to baseline activity. For 15 of the 30 remaining variants, no fold change could be calculated due to lack of baseline activity. Among the 90 non-amenable gene variants, six (A20D, A20P, L21P, V164G, G261V, and G271C) had a fold increase >1.5, but did not comply with the 5% threshold for minimal enzyme activity (see Table A2, Appendix A). Notably, 86.7% of the non-amenable variants had baseline enzyme activity <1%, another 5.6% showed enzyme activity >50%, and only 7.8% an intermediate enzyme activity between 1% and 50%. The amenable variants showed a different profile. Only 14.8% had baseline enzyme activity <1%, 68.2% had intermediate enzyme activity, and 17.0% had enzyme activity >50% baseline activity. The high percentage of variants with high baseline enzyme activity >50% is especially important as these patients should be carefully evaluated in an initial clinical examination as to whether chaperone therapy is appropriate, e.g., if sufficient evidence is available that the mutation is causal for the symptomatology. This is particularly delicate if certain outcome measures are not available to assess the success of the therapy. A different distribution was also observed for the clinical phenotype of the non-amenable as compared to the amenable variants. In total, 73.3% (66/90) of the non-amenable variants were associated with the classical phenotype, whereas the percentage of classical variants within the amenable group was only 51.1% (45/88) (see Table A2, Appendix A).

### 2.2. Inter-Assay Comparison of In Vitro Enzyme Activity between In-House and GLP Assay Data

**Figure 1:**
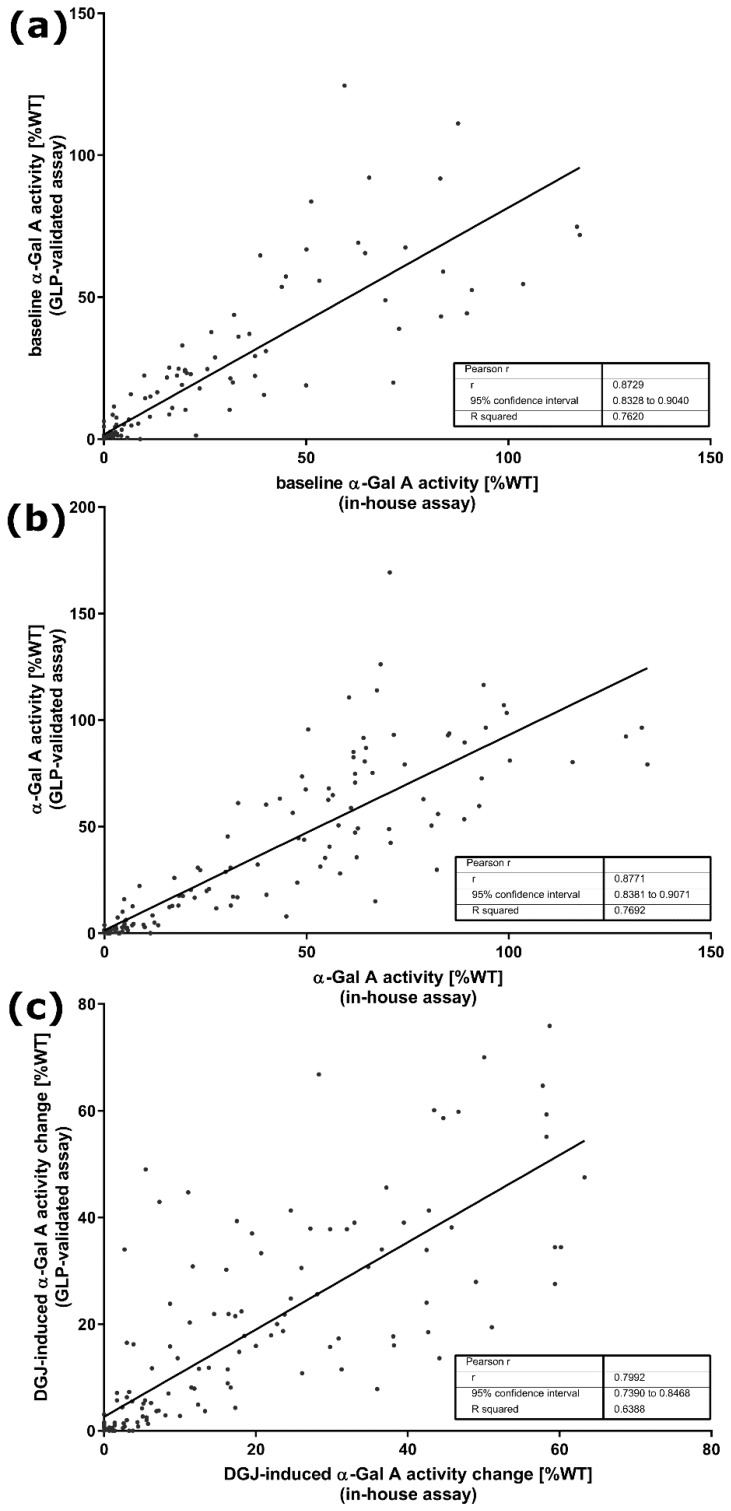
Linear correlation analysis of α-galactosidase A (α-Gal A) activity presented as absolute increase (%WT) of *GLA* variants (**a**) without and (**b**) with DGJ between the in-house assay and the GLP-validated assay. (**c**) Linear correlation analysis of DGJ-induced α-Gal A activity change over baseline (%WT) of the *GLA* variants.

**Figure 2:**
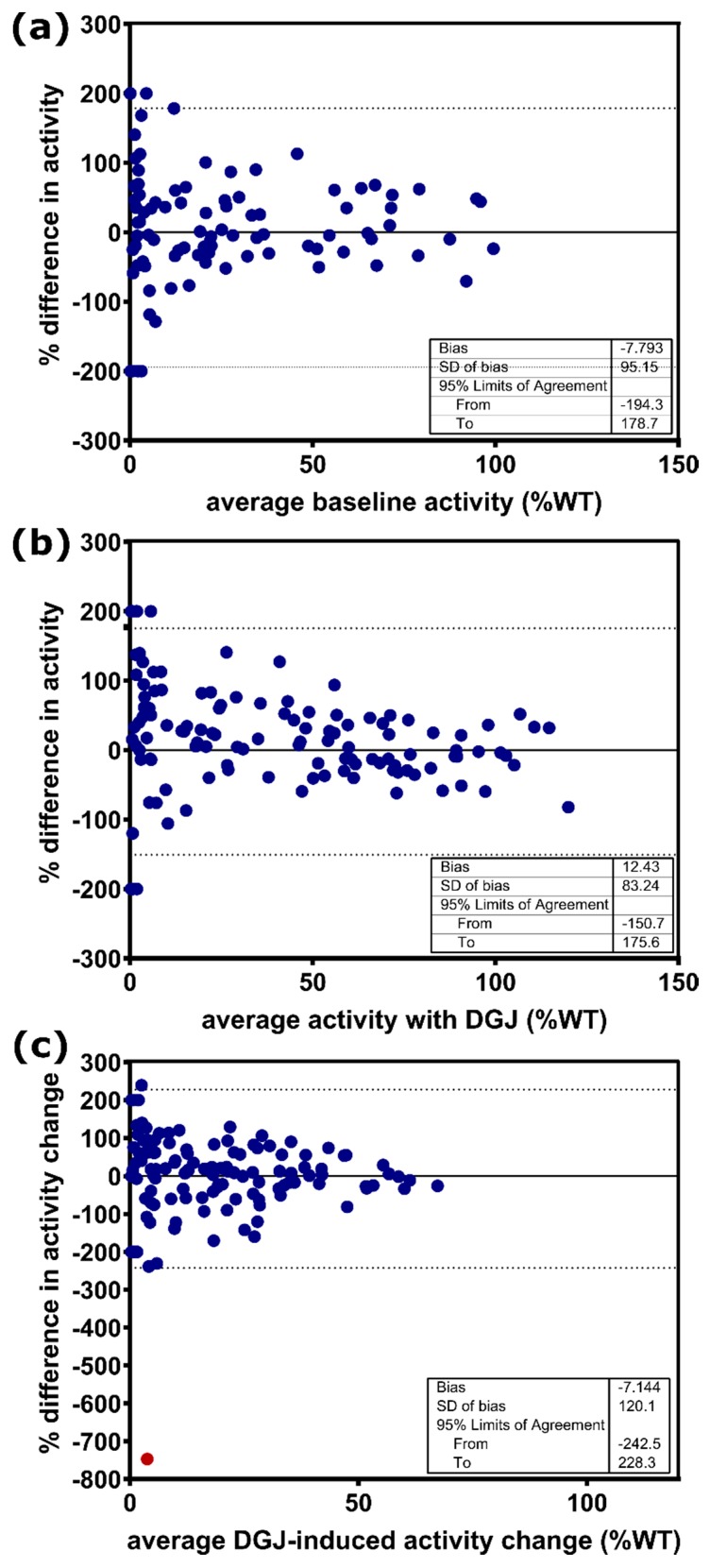
Bland–Altman analysis of α-Gal A activity for *GLA* variants expressed as percentage difference in activity (%WT) between the in-house assay and the GLP-validated assay. (**a**) Baseline α-Gal A activity without the addition of DGJ. (**b**) α-Gal A activity with DGJ. (**c**) DGJ-induced α-Gal A activity change. The dotted line indicates the 95% limit of agreement. SD: standard deviation.

**Figure A1:**
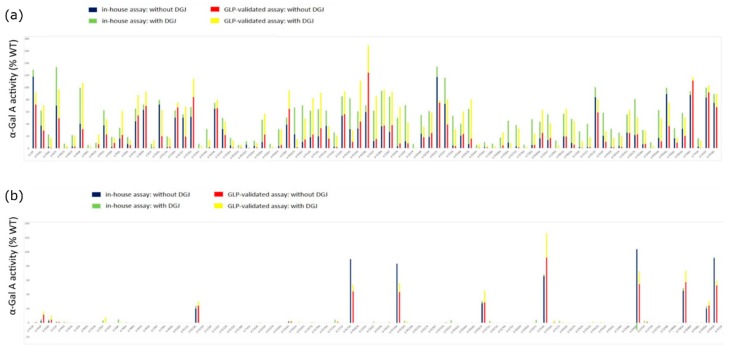
Comparison of α-Gal A activity of *GLA* gene variants without and with the addition of DGJ between the in-house assay and GLP-validated assay [14]. (**a**) *GLA* gene variants that were classified as amenable according to in-house data. (**b**) *GLA* gene variants that were classified as non-amenable according to in-house data. *GLA* variants were ordered with respect to their positions on the amino-acid sequence. Variants with no associated bar had no quantifiable α-Gal A activity without or with the addition of DGJ.

Despite some differences in design parameters between the in-house measurements and the GLP-validated study, especially concerning cultivation time and concentration of the pharmacological chaperone DGJ, both assays were designed to test the in vitro responsiveness of mutations and predict the responsiveness of patients. Table 1 shows the differences between the two assays under investigation here. We compared α-Gal A activity of all 178 *GLA* gene variants, presented as a percentage of WT (%WT) activity, without and with the addition of DGJ (see Figure A1, Appendix A) and separated the variants initially according to whether amenability was testified (see Figure A1A and Table A2, upper section, Appendix A) or not (see Figure A1B and Table A2, lower section, Appendix A) using the data obtained in our in-house assay. It is important to note that amenability classification was strictly applied according to the protocol of the respective study as summarized together with all crucial differences between the two compared assays (Table 1); therefore, the in-house data were assessed with the corresponding amenability criteria, whereas the data from the GLP-validated assay were evaluated with the dual criteria previously described [14]. Following this evaluation there was agreement between our in-house assessment and the GLP-validated study for 155 (87.1%) of the gene variants with a balanced number of amenable (11) and non-amenable (12) variants (see Table A2, Appendix A). The baseline activity appears to have a significant effect on the classification of amenability as shown above. We used the Pearson *r* linearity coefficient to test associations between the in-house and the GLP-validated assay, which revealed a good correlation for the baseline enzyme activity (Pearson correlation coefficient *r* = 0.8729, *p* < 0.0001, Figure 1A). Moreover, a similar correlation was observed between the two datasets comparing the α-Gal A activity with DGJ (*r* = 0.9448, *p* < 0.0001, Figure 1B). We also examined the DGJ-induced α-Gal A activity change over baseline as %WT and found a Pearson *r* of 0.7992 (Figure 1C). For a better comparison of the data with the previous study from Oommen and colleagues [16], we also indicated the *R^2^* from linear regression analysis which indicated higher agreement of the data despite using different assays. We obtained *R^2^* of 0.7620, 0.7692, and 0.6388 for baseline activity, activity after DGJ treatment, and DGJ-induced activity change, respectively, compared to 0.514, 0.4019, and 0.382 for the same parameters [16]. Still, the Bland–Altman analysis was in line with the previous study demonstrating a weak inter-assay correlation with 95% limits of agreement of −194.3% to 178.7% determined for the baseline activity without DGJ (Figure 2A) and −150.7% to 175.6% with DGJ (Figure 2B). The α-Gal A activity change in %WT showed 95% limits of agreement from Bland–Altman analysis of −242.5% to 228.3% between the in-house assay and the GLP-validated assay (Figure 2C). With the exclusion of the extreme result for variant A368T (red dot in Figure 2C), the 95% limits of agreement were −197.7% to 195.3%. This analysis indicated significant disagreement in the measurement of enzyme activity depending on the examining laboratory.

### 2.3. Comparison of Amenability Classification

**Figure A2:**
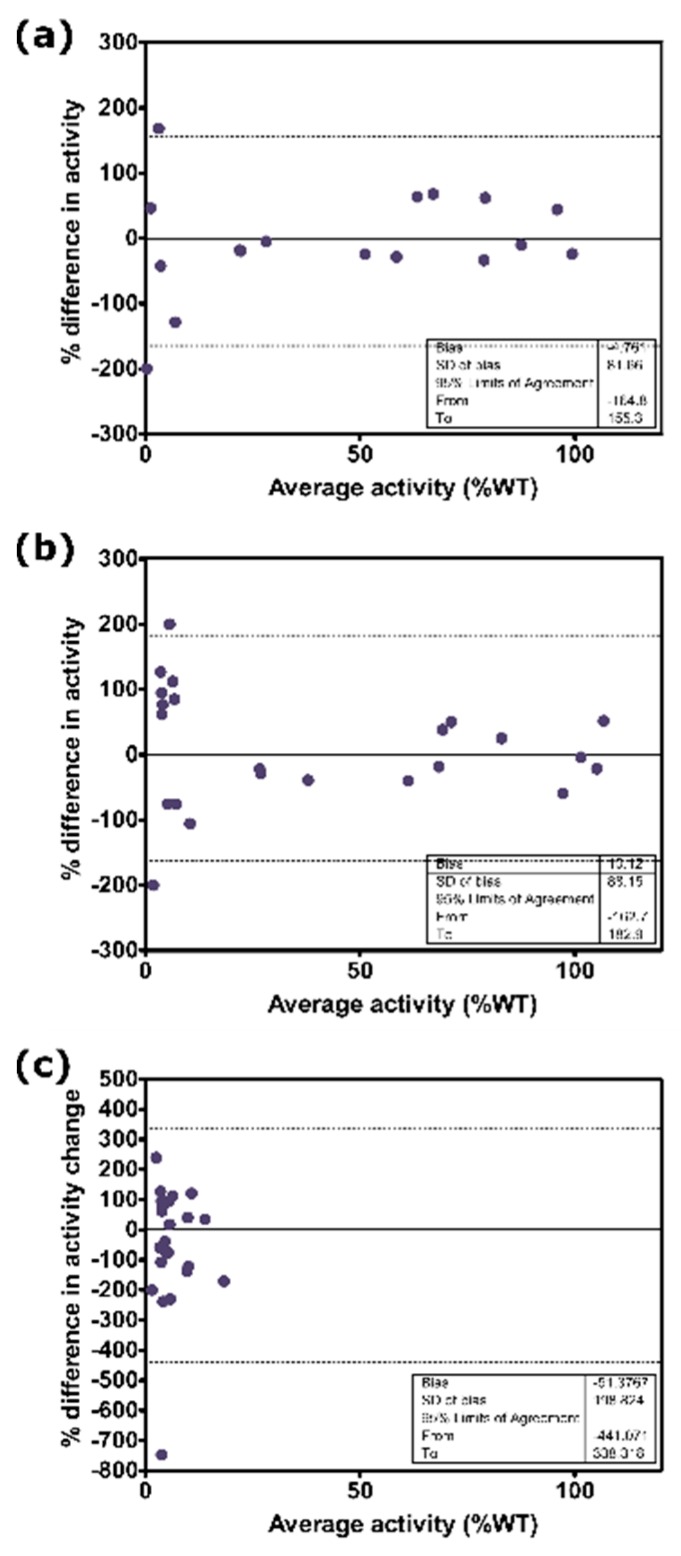
Bland–Altman analysis of α-Gal A activity for the 23 differently classified *GLA* gene variants expressed as % difference in activity (%WT) between the in-house assay and the GLP validated assay. (**a**) Baseline α-Gal A activity without the addition of DGJ. (**b**) α-Gal A activity with DGJ and (**c**) DGJ-induced α-Gal A activity change. The dotted line indicates the 95% limit of agreement. SD: standard deviation.

The relatively weak inter-assay correlation observed in the Bland–Altman plots was reinforced by the fact that the classification of amenable versus non-amenable variants was inconsistent for 12.9% (23/178) of the variants. We considered what the main risk might be for a variant classified differently. Therefore, we compared the 23 differently categorized gene variants with the remaining consistently classified variants. As observed in the former study by Oommen and colleagues [16], a high percentage of 34.8% (8/23) of the differently classified variants had high enzyme activity >50%, including D175E, K213M, R252T, V316I, A368T, F396Y, and L415F, with an essentially normal enzyme activity (min/max = 50.1%/117.9%; mean activity = 85.1%). These variants may be benign with an uncertain clinical significance. Moreover, F396Y was terminated from the Human Gene Mutation Database (HGMD) because it is not a genomic mutation [13]. Interestingly, when analyzing the isolated 23 differently classified *GLA* gene variants using Bland–Altmann analysis, the 95% limit of agreement did not differ much from the value obtained for all 178 variants: −164.8% to 155.3% (without DGJ) and −162.7% to 182.9% (with DGJ) (see Figure A2A,B, Appendix A). However, not surprisingly, for the DGJ-induced α-Gal A activity change, the Bland–Altman analysis revealed a large difference between the assays with a 95% limits of agreement from −441.1% to 338.3% (see Figure A2C, Appendix A). It is important to note that the difference in the DGJ-induced activity change of 82.6% (19/23) of the variants was higher than the applied threshold of ≥3% absolute increase from the amenability criteria of the GLP study.

### 2.4. Reconsideration of Amenability

There were 89/178 *GLA* variants classified as non-amenable according to the GLP-validated assay [14] (see Table A2, Appendix A). In total, 84/89 variants lacked the required DGJ-induced 3% increase in absolute enzyme activity, whereas 75/84 had no baseline activity and, hence, no fold over baseline value was calculated. Lastly, 6/84 showed at least the required fold over baseline activity. We tested whether a better agreement between the two datasets from the in-house assay and the GLP-validated assay could be achieved by exchanging the amenability criteria. To this end, we applied the amenability definition from the GLP-validated assay on our in-house dataset. Surprisingly, 9.6% (*n* = 17) of the variants switched categories. All switches from amenable to non-amenable (*n* = 11) were explained by an insufficient (<1.2) fold over baseline activity. Only gene variants of the category 50% activity and higher were involved (min/max = 50.0%/117.7%; mean activity = 79.3%). The cases in which the switch from non-amenable to amenable occurred (*n* = 6) could be attributed to the lower threshold of 3% absolute activity. However, the application of the different amenability definition did not lead to an improved agreement between the assays. In this analysis, 18.0% (32/178) of the variants had a discordant amenability classification with the earlier study [14], which argues for an experimental discrepancy rather than one of definition. However, when we exerted our amenability definition on the GLP-validated assay dataset, 11 variants switched category. Here, a preferred switch from amenable to non-amenable (*n* = 7) was also observed compared to the reversed direction (*n* = 4). A summary of this analysis is shown in Table 2.

The variants that failed at the fold over baseline threshold usually exhibited >50% enzyme activity and were, therefore, variants of uncertain significance, which could be associated with benign outcomes. The most frequent finding among the non-amenable variants was a non-calculable fold over baseline due to a lack of baseline activity, which, however, is not an exclusion criterion for amenability as long as the 3% threshold absolute enzyme activity is reached. Therefore, we considered the absolute %WT increase in enzyme activity to be the more relevant of the two parameters of the amenability criteria and abandoned the dual criteria in favor of a more stringent threshold for the absolute increase. We further figured that this strategy may lead to better compliance of the amenability classification. We defined common thresholds of 3%, 5%, 7%, 8%, and 10%, and then compared the data of the in-house assay and the GLP-validated assay. Interestingly, the best achievable agreement was found at a threshold of 7%. Here, only 9.0% (16/178) of gene variants were classified differently (Table 3), but the number of amenable variants was reduced to 76 or 80, depending on whether the in-house or GLP assay dataset was used. In order to achieve agreement between both datasets, the number of amenable variants was even reduced to 71. A lower set threshold or even higher threshold values also led to a slightly improved agreement compared to the use of different amenability criteria.

### 2.5. First Evaluation of DGJ amenability for 18 GLA Gene Variants

New *GLA* variants are being identified continuously, for which no treatment recommendation with DGJ can be published so promptly. We tested 18 novel *GLA* variants from the CentoMD^®^ 5.4 database [18] (CentoMD^®^ 5.4 database, queried 02/2018) for their DGJ amenability (Table 4). In total, 33.3% (6/18) of the variants had residual activity >50%, which suggests that they may have been found during differential diagnosis in patients with mild disease progression of unknown etiology [7]. Of the 18 variants, 14 were biochemically responsive to 20 µM DGJ. Eight of the 14 amenable variants met both amenability criteria, i.e., the absolute enzyme activity increase of at least 5% of WT and the 1.5-fold over baseline (D165E, F169L, G171V, M208K, P214A, Y222D, V269L, and G271A). Five of the 14 variants were classified as amenable exclusively due to the sufficient absolute increase in activity (V22A, D25V, S188A, R193S, and M208I), and for one variant the fold over baseline could not be determined due to lack of activity (G183C).

## 3. Discussion

Pharmacological chaperone therapy with the novel chaperone DGJ in Fabry disease depends on the biochemical responsiveness of the *GLA* gene variant. It was demonstrated that residual baseline activity of a gene variant has a positive effect on the likelihood of being responsive [13]; however, due to the wide range of baseline α-Gal A levels among non-amenable and amenable variants, amenability is difficult to predict and demands empirical testing. An amenability prediction method was also developed [19,20], but did not completely represent the experimental investigations [7]. We introduced a method to measure α-Gal A activity to assess the damage of *GLA* gene variants in FD [7,9,13]. A very similar method was engineered using a GLP-validated assay to predict the clinical outcome of the chaperone therapy [14]. To date, the latter assay is the only source available to obtain pivotal information on patients’ receptivity to treatment. In the present study, we compared the outcomes of the GLP-validated assay and our in-house assay. Despite experimental differences, both assays pursue the purpose of predicting patient treatment response. Amenability classification was already carried out for more than 1000 *GLA* gene variants and compiled in the current summary of product characteristics [8]. In the present study, complementary data for a subset of 178 gene variants were compared for enzymatic data and amenability classification.

Correlation analysis suggested a strong correlation of in vitro enzyme activity data between the in-house assay results and the GLP study (Figure 1). Moreover, linear regression analysis showed improved *R^2^* for baseline activity, activity after DGJ treatment, and DGJ-induced activity change as compared to the study by Oommen and colleagues [16], even though the latter study adopted the conditions of the GLP study in detail. However, this may partially be attributed to the larger number of variants investigated, because Bland–Altman analysis revealed rather strong deviation between the activity values for the individual variants in line with the former study [16]. More critically, a level of differently classified variants of 12.9% between the present study and the GLP study regarding DGJ amenability was found. However, since a higher DGJ concentration was used in combination with a shortened incubation period of 60 h in our in-house assay as compared to the GLP-validated study, one could speculate that this difference has a significant systematic impact on the reproducibility of the results. Nevertheless, it was impressively shown that even data from different cell systems (COS-7 vs. HEK293 cells) correlate very well as long as they were obtained from in vitro overexpression systems [15]. It was also reported that there was a discrepancy of 10.5% in the amenability classification [16] between a pre-GLP HEK assay developed in clinical phase II [21] and the GLP-validated study [14]. A less pronounced correlation was determined when comparing enzyme activity between overexpression systems and cells derived from patients. This finding is reflected in various clinical trial studies. The study introducing the preliminary HEK assay showed that one of eight *GLA* variants (12.5%) previously classified as amenable (F295C) failed biochemical response in DGJ-treated patients that were tested for in vivo α-Gal A activity in peripheral blood mononuclear cells (PBMCs) [21]. In another cohort, two of 16 (12.5%) variants (G144V, G325R) failed to achieve biochemical response. However, both patients showed clinical response in terms of biomarker reduction [14]; version the other hand, one patient of another cohort harboring the variant S276G showed unexpected responsiveness in the PBMC assay, but showed no reduction of biomarker. Notably, S276G is one of the variants switching category from amenable to non-amenable between References [21] and [14]. This variant is classified amenable in our in-house assay in contrast with the GLP study. All 14 patients (representing nine different *GLA* variants) in another cohort showed clinical responses in accordance with the classification of the GLP assay [14].

We hypothesized that, although the definitions of amenability appear similar, their impact on the indication of whether treatment with DGJ should be initiated may be significant. Based on the observation that many variants failed to meet the dual criteria of amenability, we considered the influence of different definitions of amenability on the observed discrepancy of 12.9% of differently classified variants. The application of the different amenability criteria to the datasets led to further inconsistencies (Table 2). Thus, we endeavored to make use of a uniform simplified amenability classification in order to achieve a better reproducibility between the assays. Since the fold over baseline criterion is invalid for many variants due to a lack of baseline activity, we based this analysis on absolute activity increase (%WT). It was assumed that the deviating classification particularly affected those gene variants that showed DGJ-induced α-Gal A in the range of the thresholds defined. Therefore, thresholds between 3% and 10% activity gain were set as a single amenability criterion. This strategy led to the conclusion that a more stringent threshold of 7% absolute activity increase led to the best compliance of the analyzed datasets with only 9.0% of the variants being differently classified (Table 3). On this basis, it could be discussed whether amenable variants that lead to a lower increase in activity should be labeled as mild or moderate responders.

To date, there is no established correlation between the biochemical enzyme activity increase induced by DGJ and the clinical benefit. Although a minimal increase in enzyme activity to 1%–6% of WT activity was suggested to be sufficient to achieve clinical benefits [22], it is highly questionable whether such an increase, observed in the in vitro cell-based assay, allows conclusions to be drawn about a beneficial outcome in vivo. It should also be considered that DGJ is an active site-specific inhibitor of α-Gal A, which may lead to total inhibition of the enzyme and worsening of the patient’s condition in gene variants with very low baseline activity. In a former study, patients with amenable *GLA* gene variants were switched from ERT to chaperone. The general result suggested that the DGJ influence on renal function and other disease-specific markers was stabilizing or even improving over the duration of the study in contrast to patients with non-amenable variants where lyso-Gb3 increased during the treatment period with DGJ [23]. In a recent study in patients with the variant N215S associated with the atypical cardiac phenotype of FD, which, to our understanding, is a strongly responsive *GLA* gene variant, an overall good outcome was shown, including increased α-Gal A activity in leucocytes and reduced plasma lyso-Gb3 [24]. However, the same study revealed that patients harboring the variant L294S, which is associated with classical FD, no baseline activity, and a moderate biochemical responsiveness of in vitro enzyme activity, did not show a beneficial outcome. This *GLA* gene variant was classified as amenable in both the GLP-validated and the in-house assay. However, the biochemical responsiveness in the GLP assay was so low that it would have been considered non-amenable according to our criteria. A recent study revealed that a patient carrying the presumed amenable variant S276N had to be switched back to ERT due to biomarker escalation [25].

It certainly remains a matter of debate whether amenability testing can still be improved by, for example, the use of *GLA* knockout cell models as recently introduced [24]. However, the cases of the variants L294S, S276G, S276N, and F295C seem to suggest that only clinical data will be able to unveil whether patients with variants of mild to moderate responsiveness will experience an equivalent benefit from the treatment as patients with strongly responding variants. Nevertheless, G325R seems to be strongly responsive in the GLP-validated assay and shows an inconsistent picture in the paraclinical data, which may be a hint that not only borderline amenable variants may show unpredictable clinical findings.

## 4. Materials and Methods

### 4.1. Material

All materials were purchased as described in the preliminary studies [7,9,13]. In brief, HEK293H cells, culture medium, all supplements, pcDNA3.1/v5-His TOPOplasmid vector, and the transfection reagent were purchased from Thermo Fisher Scientific (Carlsbad, CA, USA). Additionally, 1-deoxygalactonojirimycin hydrochloride and the synthetic fluorogenic substrate 4-methylumbelliferyl-α-d-galactopyranoside (4-MUG) for α-Gal A activity measurement were purchased from Sigma Aldrich (Steinheim, Germany).

### 4.2. Study Design and Selection of Mutations

In the present study, α-Gal A enzyme activity data from our in-house human embryonic kidney cell-based assay were compared to the good laboratory practice (GLP)-validated assay for 178 *GLA* gene variants. The differences of the assays are displayed in Table 1. The results of the α-Gal A activity measurement for 148 variants were taken from previous studies; 114 variants were measured in References [9,13], and 34 variants were measured in Reference [7]. The previously published variants M42V, N139S, G183V, N215S, L268S, L310F, S345P, R356Q, G360C [13], and S247P [7] were reassessed for the current study. Further variants A15P, W162C, D170H, G183A, M187R, E203K, P205T, Y207C, P214S, Y216C, W226R, A230T, I239T, Q250P, N263S, P265S, G271C, G271D, G274S, and M284V were selected from the CentoMD database [18] (CentoMD^®^ 5.4 database, queried 02/2018). Nonsense variants and variants where no enzyme activity was published from the GLP-validated reference assay were excluded from the study.

### 4.3. Generation of Novel GLA Mutations

The plasmid vectors containing the mutant *GLA* complementary DNA (cDNA) were produced inpcDNA3.1/v5-His TOPO using site-directed mutagenesis PCR and were analyzed according to our previous protocols [7,13].

### 4.4. In-House α-Gal A Activity Assay

The α-Gal A activity was measured as described previously [7,13]. In brief, HEK293H cells were harvested in High Pure Water (TKA Wasseraufbereitungssysteme GmbH, Niedererlberg, Germany) and lysed using the freeze and thaw method. The protein content of each sample was determined using the bicinchoninic acid (BCA) Assay Kit (Thermo Fisher, Braunschweig, Germany). Enzyme activity was measured in a sample containing 0.5 µg of total protein using the fluorogenic substrate 4-MUG. The lysates of each well were measured in duplicates in a plate reader (Tecan AG, Männedorf, Switzerland) at 360 and 465 nm, as the excitation and emission wavelength, respectively.

### 4.5. Enzyme Activity Calculation

In each experiment, the measured variant enzyme activity was corrected for endogenous enzyme activity by subtracting the average activity obtained from two wells containing pcDNA3.1/v5-His TOPOvector-only transfected cells. Enzyme activity was normalized to WT-*GLA* vector-transfected HEK293H cells (%WT) from corresponding experiments. Absolute increase in α-Gal A activity was calculated by subtracting untreated (baseline) activity from the activity after addition of 20 µM DGJ as %WT. Relative enzyme activity was determined as fold increase above baseline. Endogenous α-Gal A enzyme activity in pcDNA3.1/v5-His TOPOvector control-transfected HEK293H cells was 137.3 ± 12.0 nmol 4-MU∙mg protein^−1^∙h^−1^ without and 173.1 ± 11.8 nmol 4-MU∙mg protein^−1^∙h^−1^ (mean ± SD) with the addition of 20 µM DGJ. Wild type enzyme activity was 7333.1 ± 734.0 nmol 4-MU∙mg protein^−1^∙h^−1^ and 7985.7 ± 768.4 nmol 4-MU∙mg protein^−1^∙h^−1^ with and without DGJ, respectively.

### 4.6. Statistical Evaluation

Correlation and Bland–Altman analyses were calculated using GraphPad Prism, version 5.01.

## 5. Conclusions

The pharmacological chaperone DGJ is the model of an experimental drug. It provides highly reproducible data in different in vitro systems for assessing the amenability of different *GLA* gene variants. Treatment with the DGJ relies on biochemical responsiveness of the gene variant underlying the disease. Therefore, the genetic profile of the patients will be an essential feature for future assessment of the evaluation of treatment success with DGJ. The measurement of the responsiveness to DGJ in in vitro cell-based assays is currently a method that has no alternative for determining amenability. In comparisons of inter-assay reproducibility, a certain variability of the results of enzyme activity and the amenability classification can virtually not be prevented. An accurate appraisal should be taken into account for a treatment decision with DGJ especially in cases of low baseline activity, borderline biochemical responsiveness, and inter-assay discrepancy as risk factors to misinterpret the potential of a *GLA* gene variant to be amenable to DGJ treatment. We also recommend a very close monitoring of the patient’s well-being and biomarkers, especially lyso-Gb3 to monitor treatment response in patients.

## Figures and Tables

**Table 1:** Comparison of the good laboratory practice (GLP)-validated assay and the in-house assay.

Parameter	Good laboratory practice (GLP) Assay	In-House Assay
Cell culture	GripTite™ HEK293 MSR	HEK293H
Assay format	96 well	24 well
Transfection reagent	Fugene HD	Lipofectamine 2000
Incubation time	120 h	60 h
1-Deoxygalactonojirimycin (DGJ) concentration	10 µM	20 µM
Cell lysis condition	Lysis buffer containing 0.5% Triton X-100	Freeze and thaw in High Pure Water
Plasmid vector system	pcDNA6/v5-His A	pcDNA3.1/v5-His TOPO
Number of measurements	*n* = 5, quadruplicate	*n* ≥ 3, duplicate
Criteria for amenability	≥3% absolute increase of wild type (%WT) AND 1.2-fold increase relative to baseline α-galactosidase A (α-Gal A) activity	≥5% absolute increase (%WT) OR 1.5-fold increase relative to baseline α-Gal A activity plus a minimum of 5% activity (%WT)

**Table 2:** Impact of the different amenability definitions on *GLA* variant classification.

Reference *GLA* Variant Amenability Classification from the GLP Study [14] Was Compared to Amenability Classification Obtained Using	Number (%) of *GLA* Variants Classified Differently from the GLP Study; *n* = 178
In-house assay and amenability criteria from in-house study [13]	23 (12.9)
In-house assay and amenability criteria from the GLP-validated study [14]	32 (18.0)
GLP-validated study and amenability criteria from [13]	11 (6.2)

**Table 3:** Effects of different thresholds for absolute enzyme activity increase (%WT) as the only criterion for defining amenability of *GLA* variants when comparing data from the in-house assay and the GLP-validated assay.

Absolute Increase in α-Gal A Activity (%WT) to Define DGJ Amenability	Number (%) of *GLA* Variants Classified Differently between the GLP-Validated Assay and the In-House Assay; *n* = 178
3%	19 (10.7)
5%	22 (12.4)
7%	16 (9.0)
8%	17 (9.6)
10%	20 (11.2)

**Table 4:** Enzyme activity and DGJ amenability classification of 18 novel *GLA* gene variants.

Amino Acid	cDNA	In Vitro Enzyme Activity (%WT) in Mean ± SEM		Absolute Increase (%WT)	Fold over Baseline	Amenable According to Present Study
		Without DGJ	With DGJ			
p.L16R	c.47T > G	0.0	0.0	0.0	n/c	No
p.V22A	c.65T > C	33.2	43.3	10.2	1.3	Yes
p.D25V	c.74A > T	110.0	128.0	18.0	1.2	Yes
p.D165E	c.495T > G	7.8	19.2	11.4	2.5	Yes
p.F169L	c.505T > C	47.7	76.8	29.1	1.6	Yes
p.G171V	c.512G > T	3.6	17.0	13.4	4.8	Yes
p.G183C	c.547G > T	0.0	9.7	9.7	n/c	Yes
p.S188A	c.562T > G	91.5	116.1	24.6	1.3	Yes
p.R193S	c.579G > C	48.7	62.5	13.8	1.3	Yes
p.M208K	c.623T > A	2.7	49.6	46.9	18.5	Yes
p.M208I	c.624G > A	45.6	62.5	16.9	1.4	Yes
p.P214A	c.640C > G	63.9	130.6	66.7	2.0	Yes
p.Q221H	c.663G > C	63.2	67.1	3.9	1.1	No
p.Y222D	c.664T > G	5.2	45.9	40.7	8.9	Yes
p.F248S	c.743T > C	108.2	90.4	−17.9	0.8	No
p.D255E	c.765T > A	79.8	77.2	−2.6	1.0	No
p.V269L	c.805G > C	2.8	48.8	46.0	17.6	Yes
p.G271A	c.812G > C	12.2	60.1	47.8	4.9	Yes

Green: Result complies with in-house amenability criterion. Red: Result does not comply with in-house amenability criterion.

## Abbreviations

α-Gal A	α-galactosidase A
DGJ	1-deoxygalactonojirimycin
ERT	enzyme replacement therapy
FD	Fabry disease
GLP	good laboratory practice
HEK	human embryonic kidney cell
PBMC	peripheral blood mononuclear cells

## Appendix A

**Table A1:** Reexamination of selected *GLA* variants.

Amino Acid	cDNA	Mean In Vitro Enzyme Activity as %WT (Initial Study Result [7,13])		Absolute Increase as %WT	Fold over Baseline	Amenable According to Present Study	Category Switch Compared to Initial Study
		No DGJ	20 µM DGJ				
p.M42V	c.124A > G	0.0 (0)	7.2 (11.9)	7.2	n/c	Yes	No
p.N139S	c.416A > G	64.6 (147.8)	74.3 (176.4)	9.7	1.1	Yes	No
p.G183V	c.548G > T	0.0 (0)	6.9 (6.7)	6.9	n/c	Yes	No
p.N215S	c.644A > G	36.7 (39.5)	61.5 (63.9)	24.9	1.7	Yes	No
p.S247P	c.739T > C	0.0 (0)	6.8 (5.8)	6.4	n/c	Yes	No
p.L268S	c803T > C	0.0 (0)	7.4 (10.8)	7.0	n/c	Yes	No
p.L310F	c.928C > T	1.6 (0)	27.7 (4.1)	17.7	13.4	Yes	Yes
p.S345P	c.1033T > C	0.0 (0)	9.7 (13.3)	9.5	n/c	Yes	No
p.R356Q	c.1067G > A	33.3 (89.1)	66.3 (99.4)	33.0	2.0	Yes	No
p.G360C	c.1078G > T	16.2 (11.9)	32.9 (26.5)	15.4	2.2	Yes	No

Green: Result complies with in-house amenability criterion. Red: Result does not comply with in-house amenability criterion.

**Table A2:** *GLA* variant enzyme activity and amenability table. Upper: amenable variants; lower: non-amenable variants.

**Amenable Variants**												
**Amino Acid**	**cDNA**	**Clinical Phenotype**	**Mean In Vitro Enzyme Activity as %WT (In-House)**		**Absolute Increase as %WT**	**Fold over Baseline**	**Mean In Vitro Enzyme Activity as %WT (GLP-Validated Assay [13])**		**Absolute Increase as %WT**	**Fold over Baseline**	**Amenable (In-House)**	**Amenable (GLP-Validated [14])**
			Without DGJ	With DGJ			Without DGJ	With DGJ				
p.L3P	c.8T > C	Uncertain	117.7	129	11.3	1.1	71.9	92.2	20.3	1.3	Yes	Yes
p.D33G	c.98A > G	n/a	37.4	62	24.6	1.7	29.3	70.6	41.3	2.4	Yes	Yes
p.L36W	c.107T > G	n/a	2.3	22.3	20	9.7	0.7	16.6	15.9	23.7	Yes	Yes
p.A37T	c.109G > A	Atypical	69.6	132.9	63.3	1.9	48.9	96.4	47.5	2	Yes	Yes
p.M42V	c.124A > G	Classic	0	7.2	7.2	n/c	0.5	4.3	3.8	8.6	Yes	Yes
p.M42T	c.125T > C	Classic	2.9	21.4	18.5	7.4	2.5	20.3	17.8	8.1	Yes	Yes
p.H46P	c.137A > C	Atypical	40.1	98.8	58.7	2.5	31	106.9	75.9	3.4	Yes	Yes
**p.R49C**	**c.145C > T**	**Classic**	**0**	**5.1**	**5.1**	**n/c**	**0**	**2.7**	**2.7**	**n/c**	**Yes**	**No**
p.M51K	c.152T > A	Classic	0	8.7	8.7	n/c	6.3	22.1	15.8	3.5	Yes	Yes
p.M51I	c.153G > A	Atypical	37.4	62	24.6	1.7	22.3	47.1	24.8	2.1	Yes	Yes
p.E59K	c.175G > A	Classic	2.2	18.5	16.3	8.4	8.6	17.5	8.9	2	Yes	Yes
p.P60L	c.179C > T	Uncertain	15.6	33.1	17.5	2.1	21.7	61	39.3	2.8	Yes	Yes
p.E66K	c.196G > A	Classic	6.8	18.3	11.5	2.7	4.8	12.9	8.1	2.7	Yes	Yes
p.A73V	c.218C > T	Atypical	44	64.7	20.7	1.5	53.6	86.9	33.3	1.6	Yes	Yes
p.D83N	c.247G > A	n/a	62.9	71.6	8.7	1.1	69.2	93	23.8	1.3	Yes	Yes
p.I91T	c.272T > C	Atypical	0.7	7	6.3	10	0.9	12.6	11.7	14	Yes	Yes
p.S102L	c.305C > T	n/a	71.6	78.9	7.3	1.1	19.9	62.8	42.9	3.2	Yes	Yes
p.R112H	c.335G > A	Atypical	1.6	19.4	17.8	12.1	2.6	17.4	14.8	6.7	Yes	Yes
**p.L120V**	**c.358C > G**	**Atypical**	**50.1**	**62**	**11.9**	**1.2**	**66.8**	**74.7**	**7.9**	**1.1**	**Yes**	**No**
p.A121T	c.361G > A	Classic	50	55.5	5.5	1.1	18.9	67.9	49	3.6	Yes	Yes
p.S126G	c.376A > G	Uncertain	51.3	67.4	16.1	1.3	83.7	113.9	30.2	1.4	Yes	Yes
p.A135V	c.404C > T	Classic	0	6.9	6.9	n/c	0	3.7	3.7	n/c	Yes	Yes
p.D136E	c.408T > A	Classic	0	31.3	31.3	n/c	1.4	12.9	11.5	9.2	Yes	Yes
p.N139S	c.416A > G	Uncertain	64.6	74.3	9.7	1.2	65.5	79.1	13.6	1.2	Yes	Yes
p.A143T	c.427G > A	Atypical	31.3	49.4	18.1	1.6	21.4	43.8	22.4	2	Yes	Yes
p.A156V	c.467C > T	Classic	4.3	16.8	12.5	3.9	1.2	12.8	11.6	10.7	Yes	Yes
p.W162G	c.484T > G	Classic	0	5.2	5.2	n/c	0.8	5.9	5.1	7.4	Yes	Yes
**p.W162C**	**c.486G > C**	**Classic**	**5.8**	**11.4**	**5.6**	**2**	**0.5**	**0**	**−0.5**	**0**	**Yes**	**No**
p.D165H	c.493G > C	Classic	3.4	11.9	8.5	3.5	1.3	8.3	7	6.4	Yes	Yes
p.G183A	c.548G > C	n/a	10	46.6	36.6	4.6	22.4	56.4	34	2.5	Yes	Yes
**p.G183V**	**c.548G > T**	**Classic**	**0**	**6.9**	**6.9**	**n/c**	**0**	**2.5**	**2.5**	**n/c**	**Yes**	**No**
p.M187V	c.559A > G	Classic	22.8	67	44.2	2.9	1.3	14.9	13.6	11.5	Yes	Yes
p.M187I	c.561G > A	n/a	3.1	31.2	28.1	10.1	5.1	30.7	25.6	6	Yes	Yes
p.I198T	c.593T > C	n/a	38.7	50.4	11.7	1.3	64.7	95.5	30.8	1.5	Yes	Yes
p.P205T	c.613C > A	Classic	10.2	70.4	60.2	6.9	14.4	48.8	34.4	3.4	Yes	Yes
p.P214S	c.640C > T	n/a	18.1	61.6	43.5	3.4	22.4	82.5	60.1	3.7	Yes	Yes
p.P214L	c.641C > T	n/a	19.4	64.1	44.7	3.3	33	91.6	58.6	2.8	Yes	Yes
p.N215S	c.644A > G	Atypical	36.7	61.5	24.9	1.7	15.6	35.6	20	2.3	Yes	Yes
p.Y216C	c.647A > G	Classic	2.3	25.9	23.6	11.2	2	20.7	18.7	10.4	Yes	Yes
p.I219T	c.656T > C	Atypical	53.3	85.3	32	1.6	55.8	93.6	37.8	1.7	Yes	Yes
p.N224S	c.671A > G	Classic	31.1	82.2	51.1	2.6	10.3	29.7	19.4	2.9	Yes	Yes
p.H225D	c.673C > G	n/a	32.2	60.5	28.3	1.9	43.8	110.6	66.8	2.5	Yes	Yes
p.N228S	c.683A > G	n/a	59.5	70.6	11.1	1.2	124.5	169.2	44.7	1.4	Yes	Yes
p.I232T	c.695T > C	Atypical	11.5	61.6	50.1	5.4	15	85	70	5.7	Yes	Yes
p.S238N	c.713G > A	Atypical	36	94.3	58.3	2.6	37.1	96.4	59.3	2.6	Yes	Yes
p.I239T	c.716T > C	Classic	26.6	85	58.3	3.2	37.7	92.8	55.1	2.5	Yes	Yes
p.I242N	c725T > A	Classic	3.1	49.8	46.7	16.1	7.6	67.4	59.8	8.9	Yes	Yes
p.L243F	c.729G > C	Classic	11.4	70.8	59.4	6.2	7.9	42.3	34.4	5.4	Yes	Yes
**p.S247P**	**c.739T > C**	**Classic**	**0**	**6.8**	**6.8**	**n/c**	**0**	**1.3**	**1.3**	**n/c**	**Yes**	**No**
p.N249K	c.747C > A	Classic	23.7	54.6	30.9	2.3	17.9	35.2	17.3	2	Yes	Yes
p.Q250P	c.749A > C	Classic	18.5	61	42.5	3.3	24.8	58.7	33.9	2.4	Yes	Yes
**p.R252T**	**c.755G > C**	**Uncertain**	**117**	**134.3**	**17.3**	**1.1**	**74.8**	**79.1**	**4.3**	**1.1**	**Yes**	**No**
p.I253T	c.758T > C	Classic	73	115.8	42.8	1.6	38.9	80.2	41.3	2.1	Yes	Yes
p.I253S	c.758T > G	n/a	4.4	53.4	49	12.1	3.3	31.2	27.9	9.5	Yes	Yes
p.P259R	c.776C > G	Classic	20.5	40	19.5	2	23.3	60.3	37	2.6	Yes	Yes
p.N263S	c.788A > G	Classic	6.7	64.4	57.8	9.7	15.8	80.5	64.7	5.1	Yes	Yes
p.D264Y	c.790G > T	Classic	0	5.4	5.4	n/c	0.5	6.2	5.7	12.4	Yes	Yes
**p.P265S**	**c.793C > T**	**n/a**	**1.6**	**9.7**	**8.1**	**5.9**	**1**	**3.9**	**2.9**	**3.9**	**Yes**	**No**
**p.L268S**	**c.803T > C**	**Classic**	**0**	**7.4**	**7.4**	**n/c**	**0**	**2.8**	**2.8**	**n/c**	**Yes**	**No**
p.V269M	c.805G > A	Classic	0	17.3	17.3	n/c	4.4	25.9	21.5	5.9	Yes	Yes
p.V269A	c.806T > C	Classic	9	45	36	5	0	7.8	7.8	n/c	Yes	Yes
p.G271D	c.812G > A	n/a	3.1	37.9	34.8	12.3	1.5	32.2	30.7	21.5	Yes	Yes
**p.S276G**	**c.826A > G**	**Classic**	**0**	**5.6**	**5.6**	**n/c**	**0**	**2**	**2**	**n/c**	**Yes**	**No**
p.T282I	c.845C > T	n/a	5	47.7	42.7	9.5	5.2	23.7	18.5	4.6	Yes	Yes
p.M284V	c.850A > G	n/a	16.2	43.4	27.2	2.7	25.2	63.1	37.9	2.5	Yes	Yes
p.A291T	c.871G > A	Classic	13.2	55.7	42.5	4.2	16.5	40.5	24	2.5	Yes	Yes
p.L294S	c.881T > C	Classic	0	12.4	12.4	n/c	0	4.9	4.9	n/c	Yes	Yes
p.R301G	c.901C > G	Classic	19.3	56.5	37.2	2.9	19.1	64.7	45.6	3.4	Yes	Yes
p.R301Q	c.902G > A	Atypical	8.5	48	39.5	5.6	5.5	44.5	39	8.1	Yes	Yes
p.R301P	c.902G > C	Classic	0	5	5	n/c	0	4.2	4.2	n/c	Yes	Yes
p.L310F	c.928C > T	Classic	1.6	27.7	26.1	17.3	0.8	11.6	10.8	14.5	Yes	Yes
p.L311V	c.931C > G	n/a	1.9	40.1	38.2	21.1	2	18	16	9	Yes	Yes
p.D313Y	c.937G > T	Uncertain	83.9	100.3	16.4	1.2	59	80.9	21.9	1.4	Yes	Yes
p.I319T	c.956T > C	Classic	20.2	58.3	38.1	2.9	10.3	28	17.7	2.7	Yes	Yes
p.N320I	c.959A > T	Classic	2	31.8	29.8	15.9	1.4	17.1	15.7	12.2	Yes	Yes
p.Q321H	c.963G > C	n/a	3.3	25.3	22	7.7	1.9	19.8	17.9	10.4	Yes	Yes
p.G325S	c.973G > A	Classic	25.6	55.4	29.8	2.2	24.7	62.5	37.8	2.5	Yes	Yes
p.Q327E	c.979C > G	n/a	21.5	80.9	59.4	3.8	22.9	50.4	27.5	2.2	Yes	Yes
p.G328A	c.983G > C	Classic	6.2	30	23.8	4.8	6.9	28.7	21.8	4.2	Yes	Yes
p.S345P	c.1033T > C	Classic	0	9.7	9.7	n/c	0	3.7	3.7	n/c	Yes	Yes
p.R356W	c.1066C > T	Classic	16.9	62.7	45.8	3.7	11	49.1	38.1	4.5	Yes	Yes
p.R356Q	c.1067G > A	Atypical	33.3	66.3	33	2	36.1	75.1	39	2.1	Yes	Yes
p.G360C	c.1078G > T	Classic	16.2	32.9	16.7	2	8.7	16.8	8.1	1.9	Yes	Yes
p.R363H	c.1088G > A	Classic	31.9	57.9	26	1.8	20	50.5	30.5	2.5	Yes	Yes
**p.F396Y**	**c.1187T > A**	**n/a**	**87.6**	**93.8**	**6.2**	**1.1**	**111.2**	**116.4**	**5.2**	**1**	**Yes**	**No**
p.T410I	c.1229C > T	n/a	2.3	16.1	13.8	7	0.4	12.2	11.8	30.5	Yes	Yes
**p.L415F**	**c.1243C > T**	**n/a**	**83.2**	**99.5**	**16.3**	**1.2**	**91.8**	**103.3**	**11.5**	**1.1**	**Yes**	**No**
p.E418G	c.1253A > G	n/a	74.6	89.1	14.5	1.2	67.5	89.4	21.9	1.3	Yes	Yes
**Non-Amenable Variants**												
**Amino Acid**	**cDNA**	**Clinical Phenotype**	**Mean In Vitro Enzyme Activity as %WT (In-House)**		**Absolute Increase as %WT**	**Fold over Baseline**	**Mean In Vitro Enzyme Activity as %WT (GLP-Validated Assay [13])**		**Absolute Increase as %WT**	**Fold over Baseline**	**Amenable (In-House)**	**Amenable (GLP-Validated [14])**
			without DGJ	with DGJ			without DGJ	with DGJ				
p.A15P	c.43G > C	n/a	0	0.3	0.3	n/c	0.9	1.2	0.3	1.3	No	No
**p.A20P**	**c.58G > C**	**Atypical**	**2.5**	**4.9**	**2.4**	**2**	**11.5**	**15.9**	**4.4**	**1.4**	**No**	**Yes**
**p.A20D**	**c.59C > A**	**n/a**	**2.8**	**4.5**	**1.7**	**1.6**	**4.3**	**10**	**5.7**	**2.3**	**No**	**Yes**
p.L21P	c.62T > C	Classic	0.6	1.7	1.1	2.8	1.1	1.6	0.5	1.5	No	No
p.P40S	c.118C > T	Classic	0	1.4	1.4	n/c	0	1	1	n/c	No	No
p.G43S	c.127G > A	n/a	0	0	0	n/c	0	0	0	n/c	No	No
p.L45P	c.134T > C	Classic	0	0	0	n/c	0	0	0	n/c	No	No
p.R49G	c.145C > G	Classic	0	0	0	n/c	0	0	0	n/c	No	No
p.C52W	c.156C > G	n/a	0	0	0	n/c	0	0	0	n/c	No	No
p.C56Y	c.167G > A	Classic	0	3.3	3.3	n/c	0	7.3	7.3	n/c	No	Yes
p.C63Y	c.188G > A	Classic	0	0	0	n/c	0	0	0	n/c	No	No
p.L68F	c.202C > T	Classic	0	4.5	4.5	n/c	0	0.8	0.8	n/c	No	No
p.Y86H	c.256T > C	Classic	0	0.7	0.7	n/c	0	0	0	n/c	No	No
p.Y86D	c.256T > G	Classic	0	0	0	n/c	0	0	0	n/c	No	No
p.D93Y	c.277G > T	Atypical	0	0	0	n/c	0	0	0	n/c	No	No
p.D93E	c.279C > G	Classic	0	0	0	n/c	0	0	0	n/c	No	No
p.C94Y	c.281G > A	Classic	0	0	0	n/c	0	0	0	n/c	No	No
p.C94S	c.281G > C	Classic	0	0	0	n/c	0	0	0	n/c	No	No
p.W95L	c.284G > T	Classic	0	0	0	n/c	0	0	0	n/c	No	No
p.R100T	c.299G > C	Classic	0	0	0	n/c	0	0	0	n/c	No	No
p.R112C	c.334C > T	Classic	0	0	0	n/c	0	0	0	n/c	No	No
**p.R118C**	**c.352C > T**	**Atypical**	**20**	**23.7**	**3.7**	**1.2**	**24**	**29.5**	**5.5**	**1.2**	**No**	**Yes**
p.H125P	c.374A > C	n/a	0	0	0	n/c	0	0	0	n/c	No	No
p.L129P	c.386T > C	Classic	0	0	0	n/c	0	0	0	n/c	No	No
p.L131P	c.392T > C	Classic	0	0	0	n/c	0	0	0	n/c	No	No
p.G132R	c.394G > A	Classic	0	0	0	n/c	0	0	0	n/c	No	No
p.G132E	c.395G > A	n/a	0	0	0	n/c	0	0	0	n/c	No	No
p.G138R	c.412G > A	Classic	0	0	0	n/c	0	0	0	n/c	No	No
p.T141I	c.422C > T	Classic	0	0	0	n/c	0	0	0	n/c	No	No
p.C142R	c.424T > C	Classic	0	0	0	n/c	0.4	0	−0.4	0	No	No
p.A143P	c.427G > A	Classic	0	0	0	n/c	0	0	0	n/c	No	No
p.G147R	c.439G > A	n/a	0	0	0	n/c	0	0	0	n/c	No	No
p.A156D	c.467C > A	Classic	0	0	0	n/c	0	0	0	n/c	No	No
p.V164G	c.491T > G	Classic	1.4	2.8	1.4	2	1.7	3.2	1.5	1.9	No	No
p.D165Y	c.493G > T	Classic	0	0	0	n/c	0	1.3	1.3	n/c	No	No
p.D165V	c.494A > T	Classic	0	0	0	n/c	0	0	0	n/c	No	No
p.L167Q	c.500T > A	Classic	0	0.7	0.7	n/c	0	0.6	0.6	n/c	No	No
p.D170N	c.508G > A	Classic	0	0	0	n/c	0	0	0	n/c	No	No
p.D170H	c.508G > C	Classic	0	0	0	n/c	0	0	0	n/c	No	No
p.C172G	c.514T > G	Classic	0	4.4	4.4	n/c	1.1	2.7	1.6	2.5	No	No
p.C172Y	c.515G > A	Classic	0	0	0	n/c	0	0	0	n/c	No	No
**p.D175E**	**c.525C > G**	**n/a**	**89.8**	**89**	**−0.8**	**1**	**44.3**	**53.4**	**9.1**	**1.2**	**No**	**Yes**
p.M187R	c.560T > G	Classic	0	0	0	n/c	0	0	0	n/c	No	No
p.L191P	c.572T > C	Classic	0	0	0	n/c	0	0	0	n/c	No	No
p.C202Y	c.605G > A	Classic	0	1.4	1.4	n/c	0	0	0	n/c	No	No
p.E203K	c.607G > A	n/a	0	0	0	n/c	0	0	0	n/c	No	No
p.Y207C	c.620A > G	Classic	0	1.1	1.1	n/c	0	0	0	n/c	No	No
**p.K213M**	**c.638A > T**	**Classic**	**83.4**	**82.5**	**−0.9**	**1**	**43.2**	**55.9**	**12.7**	**1.3**	**No**	**Yes**
p.H225R	c.674A > G	Classic	0	3	3	n/c	0	2	2	n/c	No	No
p.W226R	c.676T > A	Classic	0	0	0	n/c	0	0	0	n/c	No	No
p.R227Q	c.680G > A	Classic	0	0	0	n/c	0	0	0	n/c	No	No
p.R227P	c.680G > C	Classic	0	0	0	n/c	0	0	0	n/c	No	No
p.A230T	c.688G > A	Classic	0	0	0	n/c	0	0	0	n/c	No	No
p.D231N	c.691G > A	Classic	0	0	0	n/c	0.5	0	−0.5	0	No	No
p.G261V	c.782G > T	Classic	0.2	3.5	3.3	17.5	0	0	0	n/c	No	No
p.W262C	c.786G > C	Classic	0	0	0	n/c	0	0	0	n/c	No	No
p.D264A	c.791A > C	n/a	0	0	0	n/c	0	0	0	n/c	No	No
p.D264V	c.791A > T	Classic	0	0	0	n/c	0	0	0	n/c	No	No
**p.M267T**	**c.800T > C**	**Classic**	**27.5**	**30.5**	**3**	**1.1**	**28.8**	**45.3**	**16.5**	**1.6**	**No**	**Yes**
p.G271C	c.811G > T	Classic	0.2	2.7	2.5	14.8	0	0.5	0.5	n/c	No	No
p.N272S	c.815A > G	Classic	0	0	0	n/c	0	0	0	n/c	No	No
p.G274S	c.820G > A	n/a	0	0	0	n/c	0	0	0	n/c	No	No
p.L275F	c.823C > T	Classic	0	0	0	n/c	0	0	0	n/c	No	No
p.Q283P	c.848A > C	Classic	0	0	0	n/c	0	0	0	n/c	No	No
p.A285D	c.854C > A	Classic	0	0	0	n/c	0	0	0	n/c	No	No
p.S297C	c.890C > G	Classic	0	3.8	3.8	n/c	0	0	0	n/c	No	No
**p.V316I**	**c.946G > A**	**n/a**	**65.6**	**68.3**	**2.7**	**1**	**92.1**	**126.1**	**34**	**1.4**	**No**	**Yes**
**p.V316G**	**c.947T > G**	**Classic**	**0**	**0**	**0**	**n/c**	**0.7**	**3.8**	**3.1**	**5.4**	**No**	**Yes**
p.I317S	c.950T > G	n/a	0	2.7	2.7	n/c	0	0.8	0.8	n/c	No	No
p.N320Y	c.958A > T	Classic	0	0	0	n/c	0	0.6	0.6	n/c	No	No
p.Q327K	c.979C > A	Classic	0	0	0	n/c	0	0	0	n/c	No	No
p.E341K	c.1021G > A	Classic	0	0	0	n/c	0	0	0	n/c	No	No
p.E341D	c.1023A > C	Classic	0	0	0	n/c	0	1.6	1.6	n/c	No	No
p.R342Q	c.1025G > A	Classic	0	0	0	n/c	0	0.9	0.9	n/c	No	No
p.R342P	c.1025G > C	n/a	0	0	0	n/c	0	0	0	n/c	No	No
p.R342L	c.1025G > T	Classic	0	0	0	n/c	0	0	0	n/c	No	No
p.L344P	c.1031T > C	Classic	0	0	0	n/c	0	0	0	n/c	No	No
p.E358K	c.1072G > A	Classic	0	0	0	n/c	0	0	0	n/c	No	No
**p.A368T**	**c.1102G > A**	**Atypical**	**103.7**	**93.3**	**−10.4**	**0.9**	**54.6**	**72.6**	**18**	**1.3**	**No**	**Yes**
p.L372P	c.1115T > C	n/a	0	2.6	2.6	n/c	1.2	2.6	1.4	2.2	No	No
p.L372R	c.1115T > G	Classic	0	0	0	n/c	0	0	0	n/c	No	No
p.G373D	c.1118G > A	Classic	0	0	0	n/c	0	0	0	n/c	No	No
p.C378R	c.1132T > C	n/a	0	0	0	n/c	0	0	0	n/c	No	No
p.I384N	c.1151T > A	Classic	0	0	0	n/c	0	0.8	0.8	n/c	No	No
**p.T385A**	**c.1153A > G**	**n/a**	**45**	**48.9**	**3.9**	**1.1**	**57.3**	**73.5**	**16.2**	**1.3**	**No**	**Yes**
p.Q386P	c.1157A > C	Classic	0	0	0	n/c	0	0	0	n/c	No	No
p.P389L	c.1166C > T	n/a	0	0	0	n/c	0	0.6	0.6	n/c	No	No
**p.G395A**	**c.1184G > C**	**n/a**	**20.1**	**23.1**	**3**	**1.1**	**24.4**	**30.7**	**6.3**	**1.3**	**No**	**Yes**
p.S405R	c.1213A > C	n/a	91	92.7	1.7	1	52.5	59.6	7.1	1.1	No	No
p.L415P	c.1244T > C	Classic	0	0	0	n/c	0	0	0	n/c	No	No

**Bold** = different conclusions of both assays toward DGJ amenability; n/a = not analyzed; n/c = not calculated.
